# Break Out: Urogenital Schistosomiasis and *Schistosoma haematobium* Infection in the Post-Genomic Era

**DOI:** 10.1371/journal.pntd.0001961

**Published:** 2013-03-28

**Authors:** Paul J. Brindley, Peter J. Hotez

**Affiliations:** 1 Department of Microbiology, Immunology, and Tropical Medicine, George Washington University School of Medicine and Health Sciences, Washington DC, United States of America; 2 Departments of Pediatrics and Molecular Virology and Microbiology, National School of Tropical Medicine at Baylor College of Medicine, Houston, Texas, United States of America; 3 Sabin Vaccine Institute and Texas Children's Hospital Center for Vaccine Development, Houston, Texas, United States of America

*The newly completed whole genome sequence of Schistosoma haematobium, the leading cause of human schistosomiasis and an emerging co-factor in Africa's AIDS and cancer epidemics, together with rapidly advancing genetic manipulation technologies, is providing new insights that could lead to the development of a new generation of tools for eliminating this ancient scourge*.

Today, more than 90% of the roughly 200 million cases of schistosomiasis occur in Africa [1], [2], of which approximately two-thirds are caused by *Schistosoma haematobium* [3], the etiologic agent of urogenital schistosomiasis. More recently, Charles King and his colleagues have suggested that the number of cases of *S. haematobium* may be much greater than previously believed, even possibly double or triple that of earlier prevalence estimates [4]. If confirmed, urogenital schistosomiasis may represent the most common infection or even adverse health condition in sub-Saharan Africa.

Urogenital schistosomiasis results when adult female *S. haematobium* worm pairs living in the veins draining key pelvic organs, including the bladder, uterus, and cervix, release terminal-spine eggs that penetrate the tissues and are excreted in in the urine to allow propagation of the parasite life cycle. However, many schistosome eggs fail to exit the body, and come to embolize within the capillary beds of pelvic end-organs, especially the tissues of the bladder, ureters, and female and male genital organs. Here they induce granulomas and ultimately small fibrotic nodules known as “sandy patches” [5]. Numerous granulomas and sandy patches cause bladder and ureteral inflammation associated with hematuria in more than 50% of the cases [3], in addition to organ deformities associated with ureteric obstruction, secondary urinary tract and renal infections, hydronephrosis, and ultimately renal failure in millions of people living in Africa [5].

Up to 75% of girls and women with chronic *S. haematobium* infection will also experience deposition of eggs with granulomas and sandy patches on their uterus, cervix, and lower genital tract [6], [7]. The resulting female genital schistosomiasis (FGS) is associated with contact bleeding, discharge, pain on intercourse, and secondary infections and diminished fertility; it is also a source of shame and stigma [6], [7]. FGS is not a rare condition—one estimate suggested that of the estimated 70 million children currently infected with *S. haematobium*, approximately 19 million girls and women will eventually develop FGS in the coming decade [8]. However, these numbers are likely conservative estimates, such that FGS may represent one of the most common gynecological conditions in Africa.

In addition to the pathologies outlined above, the effects of chronic *S. haematobium* infection in children have been linked to chronic inflammation and pain, as well as growth stunting and cognitive deficits among tens of millions of children [4]. FGS has also been identified as a co-factor in Africa's AIDS epidemic, especially in Zimbabwe and Tanzania, where it was linked to a 3–4 times increased risk in acquiring HIV infection [6]–[8]. *S. haematobium* eggs have now been further identified as a Group 1 carcinogen responsible for a unique squamous cell carcinoma, which is widespread in *S. haematobium*–endemic areas [5], [9]. *S. haematobium* also exerts important host endocrine effects based in part on its ability to synthesize and release estradiol [9]. A full consideration of these and other chronic morbidities that use disability-adjusted life years (DALYs) as a metric suggests that chronic urogenital schistosomiasis may equal or even exceed malaria or other better-known conditions in terms of its disease burden in Africa [4].

Despite its overwhelming public health importance and its well-established links to HIV/AIDS and cancer, *S. haematobium* has been labeled “the neglected schistosome” [10], [11]. Indeed, as shown in Table 1, a search of the literature named in PubMed (the online database of the US National Library of Medicine) supports this assertion. Over the last five years, there have been 644 papers listed in PubMed per million cases of Asian schistosomiasis caused by *Schistosoma japonicum* and 25 papers per million cases of *S. mansoni* infection, Africa's other major schistosome. In contrast, only three papers per million cases of *S. haematobium* infection have appeared in PubMed over the last five years. (The 342 publications over the past five years investigating *S. haematobium* and schistosomiasis haematobia represent challenging research; and much effort went into studies on this organism during the several decades before that. Much of this work was undertaken with humans as the host, compounding the challenges for the research and encumbering progress. Although a review of this literature is beyond the aim of our editorial, studies such as those by Agnew, Cheever, and Doenhoff on rodent models of infection [12], [13]; Capron and co-workers on vaccines [14]; Feldmeier, Kjetland, Shiff, and others on female genital schistosomiasis and bladder cancer [7], [9], [15]; Brooker, Deelder, Hamburger, Mutapi, Sturrock, and others on epidemiology, co-infections, and diagnostics [3], [16]–[18]; and Kitron, Loker, and Rollinson on intermediate host snails, ecology, and evolutionary aspects [10], [19], [20] have provided crucial scaffolds, the “shoulders of giants” as it were. Investigators accessing the newly available genetic tools can now gaze from these shoulders and hopefully move rapidly to new interventions for *S. haematobium* infection. [We apologize to investigators we did not have space to mention.])

**Table 1:** Number of citations in PubMed over the last five years, 2008–2012.

Parasite Species	Approximate Number of Human Cases	Number of PubMed Citations over the Last Five Years^b^	PubMed Citations per Millions of Human Cases	References
***Schistosoma japonicum***	1 million	644	644	Steinmann et al. 2006 [1]
***Schistosoma mansoni***	54 million^a^	1,371	25	Van der Werf et al. 2003 [3]
***Schistosoma haematobium***	112 million^a^	342	3	Van der Werf et al. 2003 [3]

a Sub-Saharan Africa only.

b Search conducted on July 14, 2012.

Nonetheless, the field of *S. haematobium* research, in contrast to studies for the other schistosomes, has largely remained “on the outside looking in” with respect to breakthroughs in the areas of omics, high throughput drug screening, reverse vaccinology, molecular imaging, bioengineering, structural biology, and molecular immunology. Whereas some of the dearth in scientific activity and productivity for urogenital schistosomiasis can be ascribed to the fact simply that it is a neglected tropical disease, this observation cannot explain why research on *S. japonicum* and *Schistosoma mansoni* has been more active and fruitful. It is plausible that the following factors are responsible for this situation: 1) the absence of mouse animal models; 2) the absence of in vitro systems; and 3) the absence of a completed genome projects and related omics projects.

The good news is that studies conducted within the past two years suggest that many of these barriers have now been surmounted either completely or partially and that there is a path forward to facilitate a new era of *S. haematobium* research.

1. *Animal models of infection*. The availability of mouse models for *S. mansoni* and S. *japonicum* infections has stimulated at least two generations of molecular and cellular immunologists to undertake groundbreaking studies on these two helminthiases and to unravel the complexities of schistosome-mediated immunopathogenesis [21]–[23]. Within the last two years (2011–2012), key studies have been conducted in genetically modified knock-out mice to also identify co-factors, leading to improved understanding of *S. haematobium* carcinogenesis [24], while microinjection of *S. haematobium* eggs in the bladder of mice has been shown to reproduce urinary tract fibrosis and other bladder histopathologic changes typically found in human urogenital schistosomiasis [25]. Expansion of such studies, including work in humanized mouse models, will undoubtedly produce new insights into parasite-induced immunopathogenesis, carcinogenesis, FGS, and HIV/AIDS co-infections.
2. *In vitro systems*. The first report of the genetic manipulation of *S. haematobium* by short interfering ribonucleic acids (siRNAs) into parasite eggs, and subsequent culturing of eggs, miracidia, schistosomula, and adult schistosomes [11], portend enormous promise for analyzing novel *S. haematobium* genes. Such in vitro studies would complement ongoing in vivo projects in mice as outlined above. Simultaneously, work in mammalian cell lines and transgenic schistosomes will provide additional insights into parasite-mediated carcinogenesis [26], [27].
3. *Omics*. In 2012, the 385-Mb genome of *S. haematobium* was completed at 74-fold coverage [28]–[30]. While there was a high synteny between the roughly 13,000 genes of *S. haematobium* and the other two schistosomes, a number of unique *S. haematobium* genes were identified, including genes encoding novel drug targets, estrogen-synthesizing enzymes that might be involved in neocarcinogenesis of eggs in situ, and molecules with immunomodulatory functions.

It is particularly promising and of great importance that the development of mouse models for *S. haematobium* infections is maturing roughly in parallel with the development of in vitro technologies for parasite gene manipulation together with the completion of whole genome sequencing for *S. haematobium*. Assuming additional proteomic and metabolomics projects for *S. haematobium* move forward, together with epigenetic studies and advances with in vitro methods linked to RNAi and transgenesis, the next few years could become a watershed for *S. haematobium* research. In so doing, we can anticipate striking new insights into parasite-induced carcinogenesis and HIV/AIDS co-infections that result from urogenital schistosomiasis.

Breakthroughs in *S. haematobium* studies will require international cooperation and a new level of support. The community of *S. haematobium* researchers is not numerically large, but could easily embrace scientists working on other schistosomes as well as those involved in cancer and AIDS research. One way to proceed is to establish a consortium of *S. haematobium* investigators and projects, possibly through joint funding from the US National Cancer Institute, National Institute of Allergy and Infectious Disease (NIAID) and/or other institutes or centers of the National Institutes of Health (NIH), the Wellcome Trust, European Union and government support, and possibly private donors such as the Bill & Melinda Gates Foundation. We believe that no one agency will likely do it all in terms of significantly advancing the *S. haematobium* research and development agenda. We also note that, with taxpayer-funded support from the NIAID-NIH, an Egyptian strain of *S. haematobium* and its intermediate host snail *Bulinus truncatus* reagents are available to interested investigators anywhere in the world from the BEI Resources catalog of the American Type Culture Collection [31].

In 2012, the global community responded to the call that “Africa is desperate for praziquantel” [32], with an unprecedented commitment of praziquantel donations from Merck KgaA and a London Declaration as a first step for the elimination of schistosomiasis as a public health problem [33]. While the drug donations and a subsequent 26 May 2012 World Health Assembly resolution calling for schistosomiasis elimination [34] are essential, we feel that alone such measures will not address Africa's burden of *S. haematobium*–induced cancer nor the stealth role of *S. haematobium* in promoting Africa's AIDS epidemic. In parallel, there needs to be put in place an aggressive program of research and development that includes fundamental studies and translational approaches toward development of new diagnostics, drugs, and vaccines. Basic research conducted within the last two years has now set the stage to go that extra mile.
